# Oxygen Saturation Sample Entropy, a Novel Diagnostic Tool in Sleep Disordered Breathing

**DOI:** 10.1007/s00408-025-00864-w

**Published:** 2026-01-21

**Authors:** Amar J. Shah, Nawal Alotaibi, Maggie Cheung, Rodanthe Nixon, Eshrina Gosal, Anita Saigal, John R. Hurst, Ali R. Mani, Swapna Mandal

**Affiliations:** 1 Respiratory, TB and Sleep Innovations Lab, UCL Respiratory, University College London, London, UK; 2 Royal Free London NHS Foundation Trust, Pond Street, London, NW3 2QG UK; 3 Network Physiology Laboratory, Division of Medicine, University College London, London, UK

**Keywords:** Sample entropy, Sleep disordered breathing, Oxygen saturation, Non-linear analysis

## Abstract

**Purpose:**

To assess whether a Network Physiology approach using Sample Entropy and Transfer Entropy can be applied to simple physiological signals obtained during sleep studies, to accurately distinguish between different types of sleep disordered breathing (SDB) and streamline the diagnostic process.

**Methods:**

Retrospective study on patients who underwent a sleep study between January 2017 and December 2021. The training dataset, used for algorithm development, included four clinically important groups: normal, obstructive sleep apnoea alone, sustained nocturnal hypoxemia with a high AHI (≥ 30/hr) and sustained nocturnal hypoxemia with a low AHI (< 30/hr). Mean, standard deviation, Sample Entropy and Transfer Entropy was calculated for heart rate, respiratory rate, oxygen saturation and nasal flow for each patient. Sample entropy is a measure of signal complexity. This was validated in a separate test dataset. ROC analysis was used.

**Results:**

In the training dataset (n = 105), the Sample Entropy of the oxygen saturation signal was significantly different in patients with SDB compared to normal studies. The area under a ROC curve for predicting obstructive sleep apnoea from normal studies and sustained hypoxia with a high AHI (≥ 30events/hr) compared to sustained hypoxia with a low AHI (AHI < 30events/hr) was 0.943 and 0.785 respectively. When tested in the test dataset (n = 80), oxygen saturation Sample Entropy above 0.1456 was 100% sensitive and 60% specific in diagnosing obstructive sleep apnoea. Patients with OSA had significantly increased Transfer Entropy from HR → SpO2, RR → SpO2 and NF → SpO2; and significantly decreased Transfer Entropy from SpO2 → RR.

**Interpretation:**

Network Physiology mapping of oxygen saturation can help distinguish between different types of sleep disordered breathing and has the potential to support simplified global diagnostic pathways for sleep apnoea utilising oximetry alone.

**Supplementary Information:**

The online version contains supplementary material available at 10.1007/s00408-025-00864-w.

## Introduction

Obstructive sleep apnoea (OSA) and obesity hypoventilation syndrome (OHS) are two of the commonest types of sleep disordered breathing (SDB) that often occur concomitantly. [1] OSA has a global prevalence of almost 1 billion, [2] with a significant proportion in low- and middle-income countries (LMIC). Furthermore, it has been estimated that approximately 10% of individuals with severe obesity and OSA will have OHS. [3] The current gold-standard diagnostic overnight multi-channel sleep study with or without an accompanying arterial blood gas is costly, cumbersome and uncomfortable for patients, and impractical in LMICs. Therefore, simplified novel diagnostic pathways are required both for OSA and OHS to improve accessibility to testing, reduce health inequality and are acceptable to patients.

Overnight pulse oximeters are simple, cost effective and agreeable to patients. However, in clinical practice, the complexity of oxygen saturation signals (SpO2) are summarised by simple static metrics such as the oxygen desaturation index (ODI). [4] It has previously been shown that SpO2 exhibits a complex pattern of fluctuations which carries additional useful information about the integrity of the respiratory system. [5, 6] This can be characterised mathematically by calculating the entropy of SpO2 which is a measure of SpO2 complexity. A recent systematic review found seven previous studies have looked at the Approximate Entropy (ApEn) of oxygen saturation in patients with OSA compared to healthy controls and found that OSA patients had a high approximate entropy. [7] However, ApEn is limited by the length of the time-series. To overcome this problem Richard and Moorman (2000) defined an alternative metric termed Sample Entropy. [8] Sample entropy quantifies the unpredictability and irregularity of a signal in a nonlinear manner. It provides a measure of the underlying physiological complexity, where lower entropy values indicate a less complex or more predictable physiological signal. [5] Previous studies have shown that reduced SpO₂ entropy is associated with diminished engagement of the cardiorespiratory system. [9].

During sleep, respiration is controlled by chemoreceptors and involves a complex network of feedback loops. Characterising the information transfer between various signals, such as heart rate and oxygen saturation, may provide valuable further information on OSA and OHS. This information can be mathematically quantified by Transfer Entropy. [10].

To our knowledge, no prior study has looked at the utlity of Sample Entropy and Transfer Entropy in adults patients with OSA and OHS. Therefore, our objectives were to assess whether Sample Entropy and Transfer Entropy of commonly acquired signals, obtained during a multi-channel cardio-respiratory polygraphy (SpO2, heart rate (HR), respiratory rate (RR) and nasal flow (NF)), can accurately differentiate OSA and OHS phenotypes. By analysing complex patters of fluctuations in these commonly derived signals, we hope to develop new diagnostic pathways with simple, cost-effective and easily accessible wearable devices such overnight oximeters. We hypothesized that Sample Entropy would discriminate between different types of sleep disordered breathing and that the Transfer Entropy from respiratory rate to SpO2 would differ by the type of sleep disordered breathing.

## Methods

### Ethical approval

This was a retrospective observational study conducted at a London teaching hospital (UK) that specialises in sleep and ventilation. The study received ethical approval from the Health Research Authority in England [REC reference 22/HRA/2206].

### Training Data Set

Patients who had undergone a diagnostic cardiorespiratory polygraphy at a single institution between January 2017 and December 2021 were initially categorised into six a priori groups. This initial dataset was felt to best represent the group of patients seen regularly in our tertiary sleep clinic. Based on preliminary data from these six groups, we chose to re-group the six groups into four clinically relevant groups which formed our training data set. The preliminary data of the six groups and the initial results are illustrated in the supplementary material.

The training dataset had four clinically relevant groups:

1. Normal sleep study (reference standard).
2. OSA alone (AHI ≥ 5events/hr). This cut off was chosen as it is the current international diagnostic standard for OSA diagnosis.
3. Sustained nocturnal hypoxemia with a high AHI (AHI ≥ 30events/hr and nocturnal hypoxia (defined as spending more than 30% of the night with saturations less than 90%) with or without daytime hypercapnia).
4. Sustained nocturnal hypoxemia with a low AHI (AHI < 30events/hr and nocturnal hypoxia, with or without daytime hypercapnia).

We chose to analyse patients with sustained nocturnal hypoxia and OSA as a different group to OSA alone, as preliminary analysis of the main dataset (supplementary material) had suggested that patients with nocturnal hypoxia may represent an early form of OHS, and thus may differ with regards to their oxygen trace variability / complexity that OSA alone.

We excluded studies that had less than four hours of recording time and those with significant central sleep apnoea (defined if the overall percentage of central events > 20% total).

### Test Data Set

This data set included a different set of unselected patients who had also undergone a diagnostic cardiorespiratory polygraphy in the same time frame. Outcome-stratified quota sampling was used to allocate the first 120 patients to the training dataset and the next 80 patients to the test dataset. Patients were categorised into the same four groups as the training data set. A graphical flowchart for patient recruitment can be seen in Figure S2 (supplementary material).

### Data Extraction and Enalysis

Baseline demographic data, comorbidities, index sleep study results and positive airway pressure therapy data were collected. The data from three channels (nasal flow, heart rate and SpO2) from the index studies were extracted and resampled to 1 Hz. All the sleep studies used a Nonin oximeter™ (Nonin Xpod Oximeter, USA). The respiratory rate was derived from nasal flow. Artefact was removed and the mean, standard deviation and Sample Entropy were calculated for each time series.

Sample Entropy is a non-linear measure which quantifies the degree of randomness and regularity in a time-series. It is a measure of complexity and calculates the probability that sequences of a certain length, *m*, in a time-series, with a certain degree of tolerance, *r*, are repeated at a later point. [5, 11] A low Sample Entropy is indicative of a regular time series, while a higher Sample Entropy indicates increased irregularity and complexity. For this study, the Sample Entropy was calculated using MATLAB codes freely available and shared on PhysioNet [12, 13] with *m* set at 2 and *r* at 0.2 as previously described. [14] To analyse the data, the data was first cleaned to remove potential artefact in the following traces:

- RR – any measurement above 30 breaths per minute was assumed to be inaccurate data capture and this measurement was changed to the median RR.
- HR – any measurement below 40 beats per minute, was assumed to be inaccurate data capture, and this measurement was changed to median HR.
- SpO2 – any measurement below 50% was assumed to be inaccurate data capture, and this measurement was changed to the median SpO2.

The percentage of artefact of each trace was noted and if a recording had ≥ 10% of aberrant data, that study was not included in the final analysis, as beyond 10% artefact, entropy values become increasingly unreliable unless robust corrections are used. [15].

Transfer Entropy quantifies the information transfer between two different physiological signals. Higher Transfer Entropy indicates more information transfer. Transfer Entropy was calculated using well established MATLAB algorithms to determine the transfer of information between all four signals, thus giving a network map with 16 interactions (4 × 4). The time lag used for the calculation was 5 s (e.g., how does the previous 5 s of SpO2 affect the next 5 s of HR). This choice was based on previous studies that indicated the Transfer Entropy between cardiorespiratory signals increases with the time lag and reaches a plateau at a time lag of 5-10 s. [16].

### Statistical Analysis

The sample size for this proof-of-concept study was calculated according to the need to demonstrate significance of 5% (p < 0.05) with 90% power. Assuming the standard deviation was 50% of the mean value we needed at least 20 patients in each of the groups to show a 50% change in sample entropy. Given we had an initial six groups in total, our sample size was 120 patients.

The Shapiro–Wilk test for normality was performed and parametric data presented as mean ± SD and non-parametric data as median (IQR). Between groups comparisons were made using either a Chi-squared test (ordinal / categorical data), ANOVA (post-hoc Tukey) for continuous parametric data and Kruskal–Wallis for non-parametric data.

Linear regression analysis was used to assess the relationship between Sample Entropy of SpO2, mean SpO2 and AHI. ROC (Receiver operating curve) analysis on the test data set was used to assess the sensitivity and specificity of sample entropy to distinguish between different forms of sleep disordered breathing. Cut-off values were determined and tested with the test data set. The rationale for these thresholds is explained in the supplementary material.

All statistical analysis was conducted in MATLAB and IBM SPSS Statistical Software Package.

Further details of the methodology, and the initial results of our main dataset can be found in the supplementary material (table S1, pages 2–10). The graphical flowchart for patient recruitment can be seen in Figure S2 (supplementary material).

## Results

**Fig. 1:**
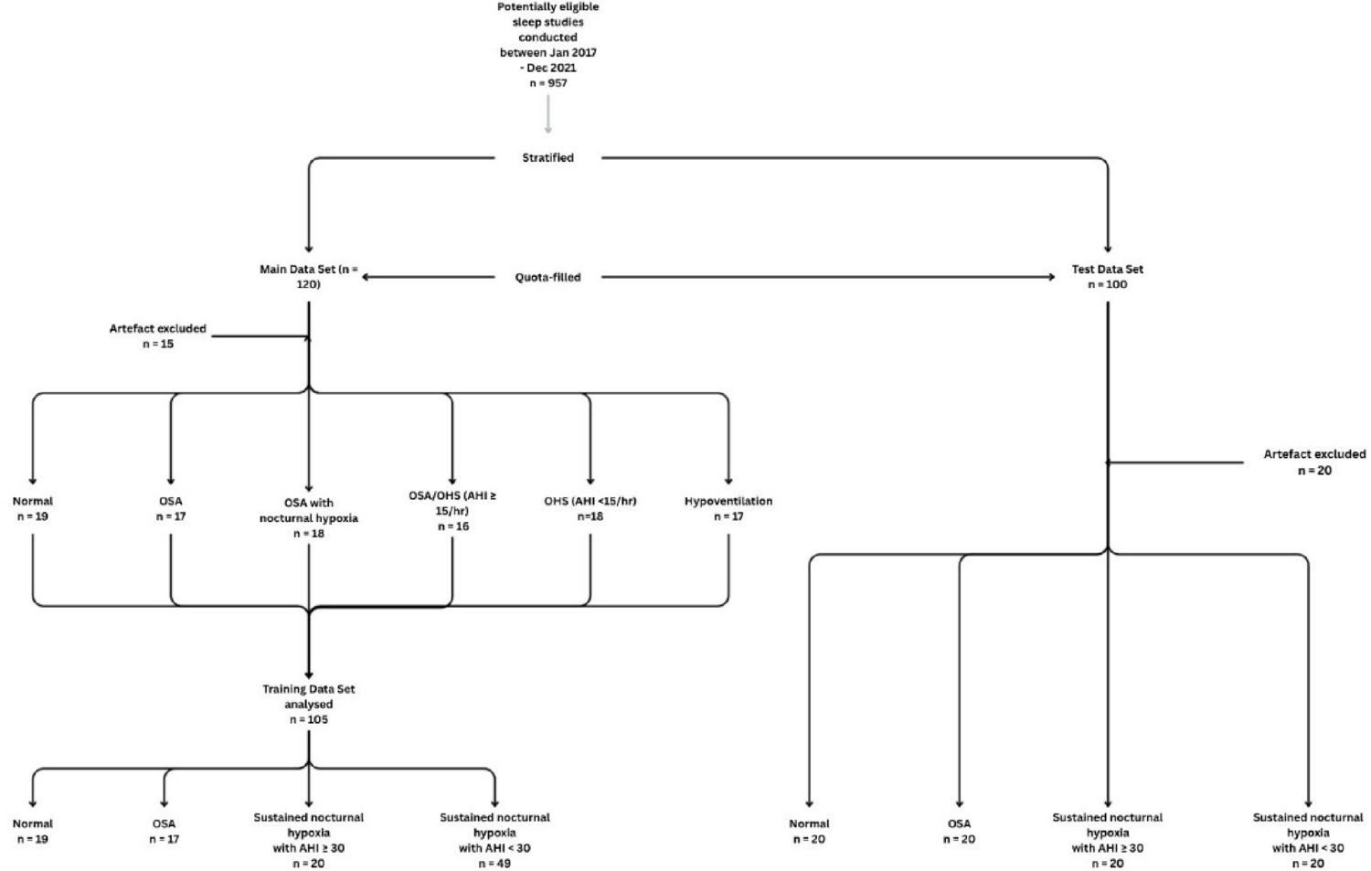
Study flow diagram

A total of 120 sleep studies in the training data set were retrospectively analysed. (Table S1, Figure S1 and Figure S2, supplementary material). Fifteen studies had significant artefact (≥ 10%) and were excluded from the final analysis. Therefore, 105 sleep studies were included in the final analysis. Baseline characteristics of the patients are reported in Table 1. The study flow diagram is illustrated in Fig. 1. Variability analysis demonstrated significant differences between the groups for the mean RR (p < 0.001), mean HR (p = 0.002), standard deviation (SD) HR (p = 0.005) and all measures of SpO2 analysis. (Table 2) Between-group differences showed that Sample Entropy of SpO2 was significantly different in all groups compared to normal sleep studies.

**Table 1:** Baseline differences between the training and test data sets

Variable	Normal Training (n = 19)	Normal Test (n = 20)	p-value*	OSA Training (n = 17)	OSA Test (n = 20)	p-value*	Hypoventilation or sustained nocturnal hypoxia with AHI ≥ 30 Training (n = 20)	Hypoventilation or sustained nocturnal hypoxia with AHI ≥ 30 Test (n = 20)	P-value*	Hypoventilation or sustained nocturnal hypoxia with AHI < 30 Training (n = 49)	Hypoventilation or sustained nocturnal hypoxia with AHI < 30 Test (n = 20)	p-value*
*Baseline demographics*												
Age at sleep time of sleep study	57 ± 14	38 ± 12	** < 0.001**	61 ± 13	54 ± 17	0.18	62 ± 13	60 ± 17	0.77	68 ± 13	64 ± 16	0.31
Female (%)	11 (58)	9 (45)	0.42	6 (32)	6 (30)	0.73	11 (55)	6 (30)	0.11	27 (55)	6 (30)	0.06
BMI	27.7 ± 5.2	28.1 ± 5.0	0.82	35.1 ± 7.5	32.3 ± 6.7	0.24	39.5 ± 9.4	37.3 ± 5.9	0.40	36.1 ± 9.3	31.7 ± 8.4	0.07
Ever- smoker (%)	6/10 (60)	7/19 (37)	0.23	6/15 (40)	9/19 (47)	0.67	7/17 (41)	12/15 (80)	**0.026**	30/43 (70)	12/19 (63)	0.61
*Medical Comorbidities*												
Atrial Fibrillation	2 (11)	0	0.14	1 (6)	0	0.27	1 (5)	4 (20)	0.15	9 (18)	0	**0.040**
COPD (%)	3 (16)	0	0.06	2 (12)	0	0.12	2 (10)	4 (20)	**0.38**	22 (45)	8 (40)	0.71
Depression (%)	2 (11)	1 (5)	0.52	0	4 (20)	0.05	6 (30)	0	**0.008**	8 (16)	2 (10)	0.50
Hypertension (%)	6 (32)	4 (20)	0.41	11 (58)	10 (50)	0.37	12 (60)	15 (75)	0.31	27 (55)	6 (30)	0.06
Hypercholesterolaemia (%)	5 (26)	3 (15)	0.38	5 (29)	6 (30)	0.97	4 (20)	3 (15)	0.68	14 (29)	0	**0.007**
IHD (%)	1 (5)	1 (5)	0.97	2 (12)	6 (30)	0.18	2 (10)	7 (35)	0.06	9 (18)	1 (5)	0.15
Kyphoscoliosis (%)	0	0	-	0	0	-	0	0	-	2 (4)	2 (10)	0.34
NAFLD (%)	0	1 (5)	0.32	0	0	-	1 (5)	1 (5)	1	3 (6)	1 (5)	0.86
MND (%)	2 (11)	0	0.14	1 (6)	0	0.27	0	0	-	4 (8)	3 (15)	0.39
Schizophrenia (%)	1 (5)	0	0.30	0	1 (5)	0.35	0	0	-	1 (2)	1 (5)	0.52
Stroke (%)	0	0	-	2 (12)	1 (5)	0.45	1 (5)	2 (10)	0.55	3 (6)	0	0.26
T2DM (%)	1 (5)	1 (5)	0.97	4 (24)	5 (25)	0.92	8 (40)	6 (30)	0.51	18 (37)	3 (15)	0.08
*Original Sleep study data*												
ESS	10 ± 5	11 ± 5	0.68	8 ± 6	11 ± 4	0.25	10 ± 7	9 ± 4	0.84	8 ± 6	10 ± 4	0.47
AHI (events/hr)	1.4 ± 1.0	1.9 ± 2.3	0.48	23.0 ± 16.3	27.4 ± 18.7	0.46	62.9 ± 25.7	49.4 ± 22.9	0.10	9.6 ± 7.9	7.7 ± 8.4	0.39
ODI (events/hr)	1.5 ± 1.1	1.9 ± 1.7	0.38	23.8 ± 15.6	28.2 ± 20.4	0.46	71.9 ± 28.0	61.9 ± 19.3	0.20	15.7 ± 12.1	12.6 ± 10.0	0.35
Mean saturations (%)	94.2 ± 1.5	94.8 ± 1.2	0.12	91.8 ± 1.1	93.3 ± 1.3	** < 0.001**	84.2 ± 5.9	86.8 ± 3.9	0.12	84.5 ± 4.9	86.1 ± 4.4	0.25
Time < 90%	1.3 ± 3.0	0.3 ± 0.7	0.19	12.6 ± 8.5	9.5 ± 6.3	0.2	75.0 ± 25.2	60.7 ± 28.3	0.10	79.1 ± 24.7	71.0 ± 34.6	0.28
*Capillary blood gas (if done)*												
pH							7.41 ± 0.4	7.42 ± 0.04	0.29	7.40 ± 0.03	7.39 ± 0.03	0.50
CO2							6.0 ± 0.8	5.8 ± 0.7	0.52	6.4 ± 0.9 ±	6.3 ± 0.7	0.83
O2							8.6 ± 1.4	8.6 ± 1.7	0.93	8.2 ± 1.2	8.4 ± 1.5	0.55
HCO3							26.6 ± 3.3	27.0 ± 4.1	0.77	27.1 ± 3.2	26.7 ± 1.9	0.64
BE							2.4 ± 3.5	3.3 ± 4.8	0.54	2.9 ± 3.3	2.6 ± 2.2	0.35

AHI, apnoea-hypopnoea index; BE, base excess; CO2, carbon dioxide; COPD, chronic obstructive pulmonary disease; ESS, Epworth Sleepiness Score; HCO3 – bicarbonate; HTN, hypertension; IHD, ischaemic heart disease; LABA, long acting beta agonist; LAMA, long acting muscarinic antagonist; MND, motor neuron disease; NAFLD, non-alcoholic fatty liver disease; NIV, non-invasive ventilation; NMD, neuromuscular disease; O2, oxygen; ODI, oxygen desaturation index; T2DM, type 2 diabetes mellitus;

^*^Most of the data was normally distributed. Continuous data presented as mean ± SD and a independent t-test was used. Categorical data was analysed using the Chi squared test

**Table 2:** Physiological variability analysis in the training data set

Variable	Normal (n = 19)	OSA (n = 17)	Sustained hypoxia with AHI ≥ 30 (n = 20)	Sustained hypoxia with AHI < 30 (n = 49)	p-value (ANOVA)
*Nasal flow (NF)*					
Mean NF (lpm)	141.30 ± 46.37	147.99 ± 68.42	149.90 ± 51.45	176.11 ± 90.75	0.24
SD NF (lpm)	177.88 ± 49.49	203.73 ± 71.65	208.21 ± 53.96	197.70 ± 78.07	0.54
Sample Entropy NF	1.621 ± 0.121	1.435 ± 0.293	1.141 ± 0.428^a,b^	1.529 ± 0.331^c^	< 0.001
*Respiratory Rate (RR)*					
Mean RR (c/m)	19.55 ± 1.78	20.00 ± 1.30	20.36 ± 1.22	21.36 ± 1.61^a,b^	< 0.001
SDRR (c/m)	4.06 ± 0.87	3.84 ± 0.79	3.89 ± 0.51	3.49 ± 0.63	0.012
Sample Entropy RR	0.958 ± 0.291	1.068 ± 0.228	1.135 ± 0.275	1.147 ± 0.262^a^	0.063
*Heart rate (HR)*					
Mean HR (c/m)	64.60 ± 10.68	70.87 ± 14.65	79.36 ± 12.55^a^	77.32 ± 14.36^a^	0.002
SDHR (c/m)	4.86 ± 1.65	5.33 ± 1.72	6.97 ± 2.45^a^	5.18 ± 2.04^c^	0.005
Sample Entropy HR	0.398 ± 0.230	0.340 ± 0.141	0.412 ± 0.162	0.351 ± 0.216	0.56
*Oxygen saturation (SpO2)*					
Mean SpO2 (%)	94.05 ± 1.30	91.75 ± 0.93	84.08 ± 5.50^a,b^	84.72 ± 4.79^a,b^	< 0.001
SD SpO2 (%)	1.50 ± 0.63	2.47 ± 0.80	5.16 ± 2.04^a,b^	3.36 ± 1.49^a^	< 0.001
Sample Entropy SpO2	0.129 ± 0.040	0.279 ± 0.108^a^	0.414 ± 0.123^a,b^	0.229 ± 0.080^a,c^	< 0.001

c/m, count per minute; lpm, Litres per minute; HR, heart rate; NF, nasal flow; SpO2, oxygen saturaton SD, standard deviation; SDHR, standard deviation of heart rate SE, sample entropy. Majority of the data was normally distributed and between group comparisons made with a one-way ANOVA. Post hoc analysis (Tukey): ^a^p < 0.05 in comparison with normal; ^b^p < 0.05 in comparison to OSA; ^c^p < 0.05 in comparison to hypoventilation with AHI ≥ 30events/hr; Sample entropy measurements have no units

Linear regression analysis with mean SpO2 and Sample Entropy SpO2 showed no significant correlations in any of the groups. There was a strong positive linear correlation between Sample Entropy SpO2 and the index AHI (r = 0.755, p < 0.001). Multiple regression analysis with gender, age, BMI and Sample Entropy SpO2 significantly predicted AHI (F(4,96) = 35.40, p < 0.001). Sample Entropy SpO2 was the only variable that added statistical significance to the prediction (β = 0.719, p < 0.001). Linear correlations were also found between Sample Entropy SpO2 and the index ODI (r = 0.770, p < 0.001) and Sample Entropy SpO2 and time below 90% (r = 0.251, p = 0.010).

**Fig. 2:**
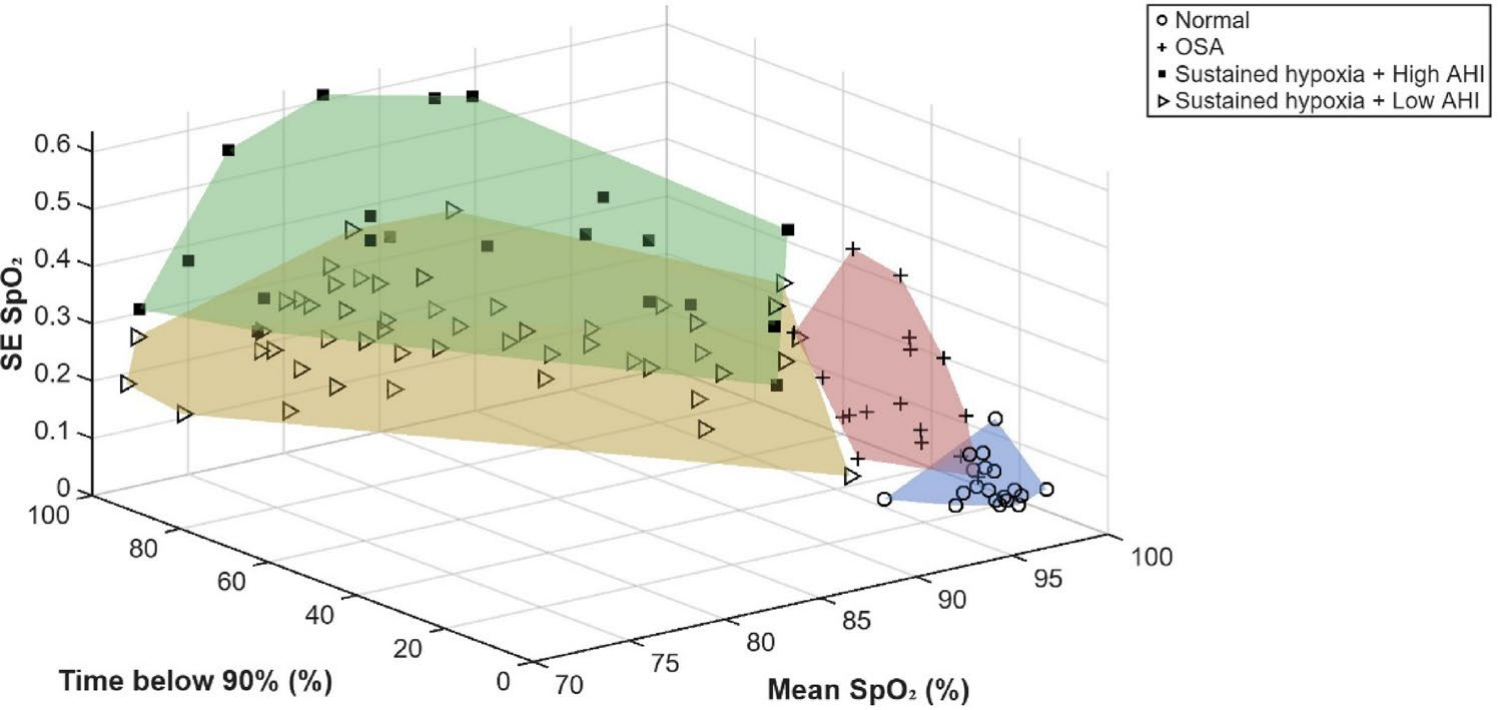
3-D graph map of the mean sats, time spent with saturations below 90% and Sample Entropy SpO2

**Fig. 3:**
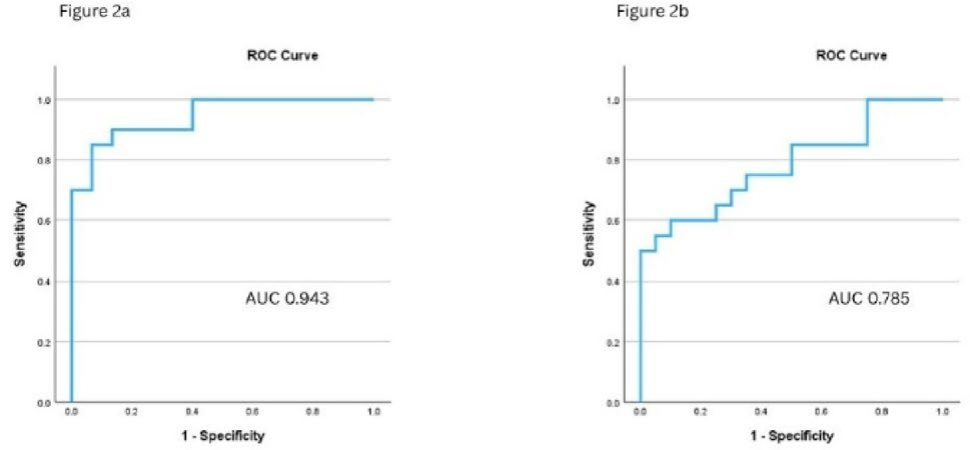
ROC analyses from the test dataset for predicting OSA from normal studies (2A); and sustained nocturnal hypoxia with a high AHI (≥ 30 events/hr) from sustained nocturnal hypoxia with a low AHI (2B)

Figure 2 maps the mean SpO2, time spent with saturations below 90% and Sample Entropy SpO2. ROC analysis from the training dataset for predicting OSA from normal studies; moderate/severe OSA (AHI ≥ 15 events/hr) from mild OSA; and sustained hypoxia with a high AHI (AHI ≥ 30 events/hr) from sustained hypoxia with a low AHI (AHI < 30events/hr) had area under curves (AUCs) of 0.950, 0.971 and 0.907 respectively. The ROC analysis from the test dataset for predicting OSA from normal studies and sustained hypoxia with a high AHI (AHI ≥ 30 events/hr) from sustained hypoxia with a low AHI (AHI < 30events/hr) had AUCs of 0.943 and 0.785 respectively. This is illustrated in Fig. 3

We subsequently developed an algorithm from the training dataset to accurately distinguish the four groups based on time spent with saturations below 90% and Sample Entropy SpO2. From the training ROC curves a Sample Entropy SpO2 ≥ 0.1456 had 100% sensitivity and 74% specificity for predicting OSA from normal studies; and a Sample Entropy SpO2 ≥ 0.3504 had 92% specificity and 70% sensitivity for predicting sustained hypoxia with a high AHI (AHI ≥ 30 events/hr) from sustained hypoxia with a low AHI (AHI < 30events/hr).

A total of 100 studies were analysed in the test dataset. Twenty studies had significant artefact (≥ 10%) and were excluded from the final analysis. Therefore, 80 sleep studies were included in the test dataset analysis. The baseline characteristics were similar to the training data set (Table 1). A Sample Entropy SpO2 ≥ 0.1456 had 100% sensitivity and 60% specificity in predicting OSA from normal. A Sample Entropy SpO2 ≥ 0.3504 had 60% sensitivity and 85% specificity for predicting sustained hypoxia with a high AHI (≥ 30events/hr) compared to sustained hypoxia with a low AHI (< 30events/hr).

**Fig. 4:**
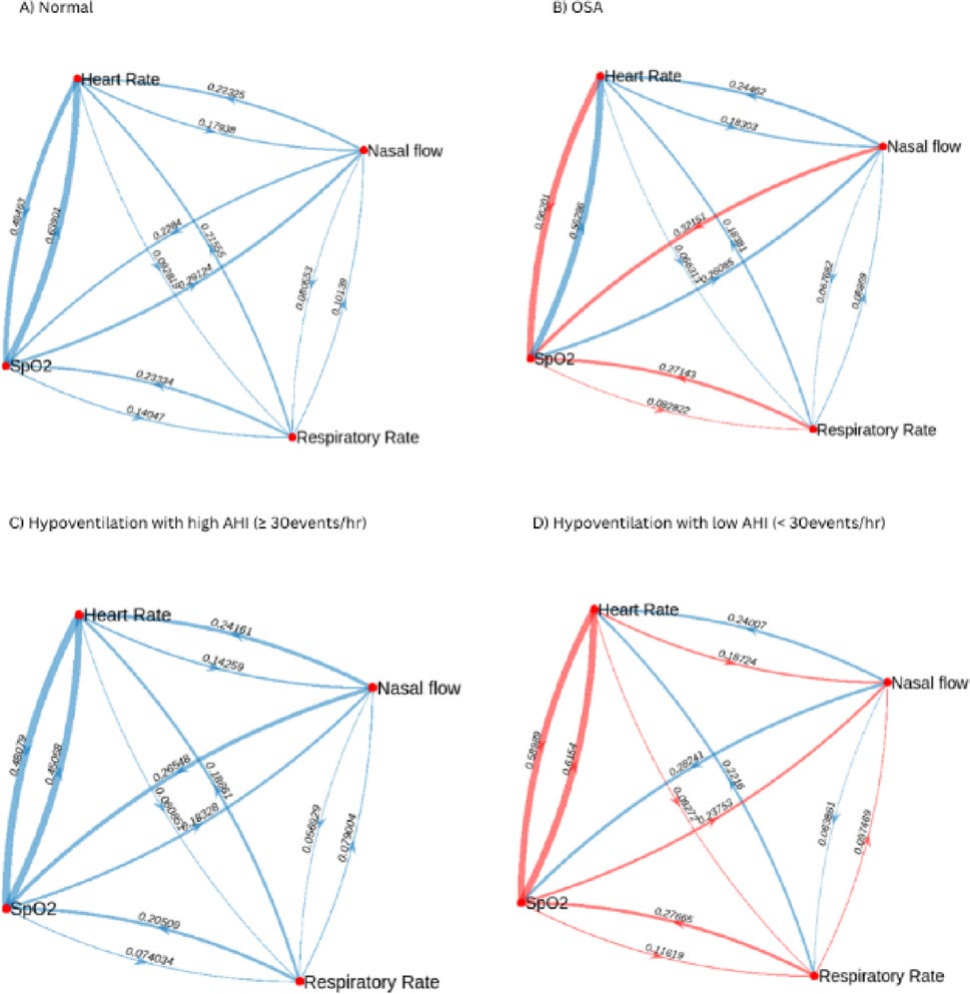
Graphical presentation of directed Transfer Entropy between different physiological parameters. Each node represents a physiological time series. Network edges (links) represent the transfer entropy with arrows indicating the direction of travel. Red highlighted edges represent a statistically significant difference between normal (A) and OSA (B) and between hypoventilation with a high AHI (C) and hypoventilation with a low AHI (D)

Graphical presentation of directed Transfer Entropy between the different physiological parameters comparing normal sleep studies with OSA is shown in Fig. 4a and b. Patients with OSA had a significant increase in Transfer Entropy for HR → SpO2; RR → SpO2 and NF → SpO2; and a significant decrease in Transfer Entropy for SpO2 → RR. There was also a trend towards decreased Transfer Entropy for HR → RR (p = 0.08) and RR → HR (p = 0.08).

Graphical presentation of directed Transfer Entropy between the different physiological parameters comparing the two groups with sustained hypoxia (AHI ≥ 30events/hr and AHI < 30 events/hr) is shown in Fig. 4c and d. Patients with AHI < 30events/hr and sustained hypoxia had a significant increase in Transfer Entropy for SpO2 → HR, HR → SpO2, SpO2 → RR, RR → SpO2, SpO2 → NF, HR → RR, HR → NF and RR → NF.

## Discussion

We show that Sample Entropy of overnight oxygen saturations has good discriminative accuracy to distinguish different forms of SDB. Sample Entropy SpO2 is simpler to measure as it only requires an oximeter compared to other diagnostic sleep disordered breathing parameters and thus has the potential to transform diagnostic pathways including in resource constrained settings including LMICs.

The significantly higher Sample Entropy SpO2 found in patients with sleep disordered breathing compared to normal sleep studies, is indicative of increased oxygen signal complexity and irregularity. This can be explained by the underlying pathophysiology of OSA, where patients have increased intermittent nocturnal hypoxia, leading to increased fluctuation of the oxygen saturation signal. Increased Sample Entropy SpO2 can also indicate enhanced engagement of physiological control system in response to hypoxia in OSA [9] which is compatible with our finding on Transfer Entropy analysis where HR → SpO2, RR → SpO2 and NF → SpO2 were higher in OSA than normal studies (Fig. 4). This is similar to previous work by Hornero et al., who found that the entropy of SpO2 was increased in patients with OSA. [17] While previous work has suggested that patients with OSA have decreased HR complexity, measured by Sample Entropy [18] and fuzzy entropy, [19] our work demonstrated no differences in the sample entropy of heart rate amongst the groups.

We found a strong positive correlation between AHI and Sample Entropy SpO2, which remained significant after adjustment for potential confounders. This suggests that patients with increasing severity of OSA have an increased Sample Entropy SpO2. This likely reflects patients with a higher AHI having increased nocturnal desaturations and thus increased complexity of the oxygen saturation signal. It also suggests that Sample Entropy SpO2 (using an SpO2 signal alone) can be used as a novel diagnostic tool in OSA.

Our test data set included four groups. Two groups had a SpO2 time below 90% of < 30% and represented either normal studies or those with OSA alone. In the other two groups, patients had a SpO2 time below 90% of ≥ 30 and thus had sustained nocturnal hypoxia. These groups were then split based on AHI. Patients with sustained hypoxia and a low AHI (< 30events/hr) had significantly lower sample entropy of SpO2 compared to those with sustained hypoxia and a high AHI. This is significant as suggests reduced system engagement, increased regularity, and therefore a sicker patient population, for whom a different treatment strategy may be necessary. ROC analysis of the test dataset using Sample Entropy SpO2 showed high sensitivity and specificity in predicting OSA from normal studies (AUC 0.943) and from sustained hypoxia with a high AHI (≥ 30events/hr), and sustained hypoxia with a low AHI (AHI < 30 events/hr) (AUC 0.785).

It is important to note that Sample Entropy SpO2 and time spent below 90% can both be calculated simply from an overnight single-channel oximetry. While many centres globally use overnight oximetry to diagnose OSA, limitations include under-diagnosis, poor performance in co-morbid patients and limited diagnostic utility in the hypoventilation population. [20] However, they are more cost-effective and easily accessible. Therefore, finding other measures that can accurately diagnose sleep disordered breathing using overnight oximetry are important. Sample Entropy SpO2 and time spent below 90% can potentially differentiate between OSA phenotypes (mild vs. moderate/severe) and OSA phenotypes with sustained hypoxia (severe vs. non-severe). While further work in this field is necessary to understand the treatment implications of this and whether this translates to a pure OHS population, this work is promising and could increase availability of accurate sleep diagnostic testing globally in a manner that is more affordable and acceptable to patients, and potentially means that patients do not have to undergo blood gas testing. Our work is suggestive that the use of sample entropy with a measure of overnight ventilation (e.g., venous bicarbonate) may pave the way to non-invasive diagnostic measures and initial treatment strategies in people with OHS with or without OSA.

From our training data set cut-off values for Sample Entropy SpO2 were chosen to represent clinical importance, for example the value chosen to distinguish OSA from normal studies was 100% sensitive, so as not to miss any potential patients with OSA. The value chosen to distinguish sustained nocturnal hypoxia with a high AHI (≥ 30events/hr) from sustained nocturnal hypoxia with a low AHI (< 30events/hr) was 92% specific, such that there were few false positives. We validated this pathway on a different set of test patients and showed that a Sample Entropy SpO2 ≥ 0.1456 had 100% sensitivity and 60% specificity in predicting OSA from normal; and a Sample Entropy SpO2 ≥ 0.3504 had 60% sensitivity and 85% specificity for predicting sustained nocturnal hypoxia with a high AHI compared to hypoventilation with a low AHI. While this needs further validation in a larger dataset, it suggests that SE SpO2 is a useful potential future metric to aid both diagnosis and management of sleep disordered breathing with easy world-wide applicability.

To our knowledge, no prior study has looked at using Transfer Entropy to create a network physiology map in patients with sleep disordered breathing. This data supports the existence of multiple feedback loops and bi-directional interactions between nasal flow, heart rate, respiratory rate, and oxygen saturation during normal sleep. This highlights that sleep is not a passive state but involves significant information transfer between different physiological time-series that are dependently linked to each other.

Patients with OSA had significantly increased directed Transfer Entropy from HR → SpO2, RR → SpO2 and NF → SpO2; and significantly decreased Transfer Entropy from SpO2 → RR. The differences in information transfer between RR and SpO2 provide further evidence of an aberrant loop gain pathway that is known to exist in OSA. [21, 22] Ventilatory loop gain reflects the ratio between the ventilatory response to the disturbance, i.e., when breathing deviates from a normal level, the response of the system matches this to ensure status quo. However, in OSA, there is higher loop gain with a disproportionately larger response. [23] This may be reflected in an increase in information transfer from RR → SpO2 (i.e., larger response) and a decrease in information transfer from SpO2 → RR (i.e., reduced acknowledgement of the respiratory disturbance). Reduced information transfer between SpO2 → RR may indicate that hypoxia may not initially increase respiratory drive in OSA and gives pathophysiologic insight about the disease process using non-invasive method. Moreover, non-invasive assessment of loop gain is not currently straightforward in clinical practice, and our findings suggest that a Network Physiology approach has a potential application in assessment of loop gain function for individual patients, which can potentially lead to individualised treatment pathways in the future. Network physiological mapping could also lead to non-invasive endotyping of OSA which will also have an impact on treatment decisions and potentially open the door to other treatment strategies including medication.

Patients with sustained nocturnal hypoxia and non-severe OSA had significant increases in their directed Transfer Entropy nearly across the whole network map. This suggests a system that is stressed with increased information exchange. This provides further evidence that this patient group is likely to represent a more severe disease process with increased morbidity and mortality. Furthermore, Transfer Entropy between HR and SpO2 can be calculated from an overnight oximetry and therefore add and support a diagnosis of sustained nocturnal hypoxia and non-severe OSA compared to sustained nocturnal hypoxia with severe OSA.

There are some limitations to this work. Firstly, our dataset consists of mainly a UK white population, and the conclusions need to be tested in a more diverse dataset given we know that skin pigmentation can affect oxygen saturation measurement. [24] Second, the retrospective nature of this study, makes firm conclusions difficult and future prospective studies are needed. Third, all the sleep studies used a Nonin oximeter™ (Nonin Xpod Oximeter, USA) and it is possible that different oximeters may generate different sample entropy results. Fourth, as not all patients had blood gas measurements we were unable to draw conclusions on patients with OHS based on current standard guidance.

In conclusion, we have shown that Sample Entropy of oxygen saturation along with Transfer Entropy can help distinguish between different types of sleep disordered breathing. In combination with current widely used parameters such as mean saturations and time spent with saturation below 90%, this new metric has the potential to create a new, simplified and inexpensive diagnostic pathway for sleep disordered breathing.

## Supplementary Information

Below is the link to the electronic supplementary material.

Supplementary Material 1

## Data Availability

Data is provided within the manuscript or supplementary information files.
