# Supplementary material for: Oxygen Saturation Sample Entropy, a Novel Diagnostic Tool in Sleep Disordered Breathing
Source: Lung. 2026 Jan 21;204(1):5. doi: 10.1007/s00408-025-00864-w (PMC12823762; doi:10.1007/s00408-025-00864-w)
Supplement: Supplementary file 1 — Supplementary Material 1 [file 408_2025_864_MOESM1_ESM.docx]

**Oxygen saturation Sample Entropy, a novel diagnostic tool in sleep disordered breathing**

Amar J. Shah, Nawal Alotaibi, Maggie Cheung, Rodanthe Nixon, Eshrina Gosal, Anita Saigal, John R. Hurst, Ali R. Mani, Swapna Mandal

**Online Data Supplement**

**METHODOLOGY – Supplementary material**

**Main data set**

Patients who had undergone a diagnostic cardiorespiratory polygraphy at a single institution between January 2017 and December 2021 were categorised into one of the six a priori categories (based on documented clinical judgement, previous additional investigations such as blood gases and multidisciplinary meeting reviews)

The categories were chosen as they were felt to best represent the group of patients that are seen regularly in our tertiary sleep clinic. Patients were categorised into these six groups based on prior documented clinical judgment, previous additional investigations (e.g., blood gas measurements) and multidisciplinary meetings.

Sleep studies were scored according to the American Association of Sleep Medicine guidelines, and 4% desaturation was used to quantify hypopnoeas and oxygen desaturation index as it was the standard used in 2017.

**Six categories of sleep studies**

1. **Normal –** This was the reference standard. These were level 3 sleep studies conducted between January 2017 and December 2021 in patients referred to the sleep clinic due to symptoms or suspicion of sleep disordered breathing. The subsequent sleep study was normal and ruled out sleep disordered breathing.
2. **OSA alone (AHI ≥ 5events/hr) –** This is the current, internationally recognised definition of obstructive sleep apnoea. As per our usual practice, which is line with national standards, this group of patients does not usually undergo capillary blood gas measurement. In our routine practice, we only perform capillary blood gas measurements if there is a suspicion of hypoventilation based on the overnight oxygen trace or evidence of significant nocturnal hypoxia (defined as spending more than 30% of the night with saturations less than 90%).
3. **OSA with significant nocturnal hypoxia (AHI ≥ 5events/hr and time with saturations below 90% of ≥ 30% and daytime eucapnia (PaCO2 <6kPa)) –** The 30% cut off was chosen as this is the level current used in clinical practice at our tertiary service and has previously been shown to be a predictor of daytime hypercapnia. All patients with a time below 90% of ≥ 30% will undergo a blood gas measurement to ensure they do not have hypoventilation syndrome. The 30% cut off is also an accepted international standard for significant nocturnal desaturation. (1-3)
4. **OSA/OHS (AHI ≥ 15 events/hr plus BMI ≥ 30Kg/m2 plus daytime hypercapnia (PaCO2 ≥ 6kPa).** An AHI cut off of ≥ 15events/hr was chosen as this represents clinically significant OSA. The remainder of the criteria is the standard diagnostic criteria for obesity hypoventilation syndrome (OHS).
5. **OHS only (AHI < 15events/hr plus BMI ≥ 30kg/m2 plus daytime hypercapnia (PaCO2 ≥6kPa).** This group had obesity hypoventilation syndrome with either no OSA or mild OSA (AHI < 15events/hr). Mild OSA is not thought to be clinically significant and therefore this cut off was chosen to represent a group with a ‘purer’ form of hypoventilation syndrome.
6. **Hypoventilation from another cause with or without OSA (BMI < 30kg/m2 with daytime hypercapnia (PaCO2 ≥ 6kPa) plus a known cause of hypoventilation (e.g., neuromuscular disease, COPD and chest wall disease).** This group was chosen to assess whether other causes of hypoventilation behave in a similar manner to obesity hypoventilation. They also make up a significant proportion of the patients seen in the sleep clinic.

In the preliminary analysis of this main dataset (n=120), 15 had artefact, meaning 105 were analysed. Table S1 shows the baseline characteristics of the main dataset.

Table S1: Baseline demographics of the main dataset (n = 105)

| **Variable** | **Normal**  **(n = 19)** | **OSA**  **(n = 17)** | **OSA with nocturnal hypoxia**  **(n = 18)** | **OSA/OHS (AHI ≥ 15/hr)**  **(n = 16)** | **OHS (AHI <15/hr)**  **(n=18)** | **Hypoventilation**  **(n=17)** | **p-value*** |
| --- | --- | --- | --- | --- | --- | --- | --- |
| **Baseline demographics** | | | | | | | |
| **Age at sleep time of sleep study** | 57 ± 13.8 | 61 ± 12.8 | 66 ± 11.5 | 64 ± 15.4 | 66 ± 15.7 | 67 ± 9.9 | 0.16 |
| **Female (%)** | 11 (58) | 6 (35) | 12 (67) | 8 (50) | 9 (50) | 9 (53) | 0.58 |
| **Caucasian (%)** | 17 (90) | 11 (65) | 10 (56) | 7 (44) | 12 (67) | 7 (41) | **0.037** |
| **BMI** | 27.70 ± 5.16 | 35.08 ± 7.53 | 41.46 ± 8.90 | 40.16 ± 4.60 | 40.65 ± 7.85 | 26.26 ± 5.48 | **<0.001** |
| **Ever- smoker (%)** | 6/10 (60) | 6/15 (40) | 8/16 (50) | 6/12 (50) | 13/17 (20) | 10/15 (67) | 0.35 |
| **Deprivation index** | 5.32 ± 2.8 | 4.53 ± 3.09 | 4.56 ± 2.26 | 3.87 ± 1.96 | 4.61 ± 2.59 | 5.29 ± 2.29 | 0.58 |
| **Medical Comorbidities** | | | | | | | |
| **Atrial Fibrillation** | 2 (11) | 1 (6) | 2 (11) | 4 (25) | 2 (11) | 2 (12) | 0.68 |
| **COPD (%)** | 3 (16) | 2 (12) | 4 (22) | 3 (19) | 9 (50) | 8 (47) | **0.034** |
| **Depression (%)** | 2 (11) | 0 | 3 (17) | 5 (31) | 3 (17) | 3 (18) | 0.25 |
| **Epilepsy (%)** | 2 (11) | 0 | 0 | 1 (6) | 0 | 0 | 0.24 |
| **Heart Failure (%)** | 0 | 1 (6) | 1 (6) | 2 (13) | 1 (6) | 0 | 0.55 |
| **Hypertension (%)** | 6 (32) | 11 (65) | 12 (67) | 9 (56) | 11 (61) | 7 (41) | 0.20 |
| **Hypercholesterolaemia (%)** | 5 (26) | 5 (29) | 5 (28) | 3 (19) | 8 (44) | 2 (12) | 0.37 |
| **IHD (%)** | 1 (5) | 2 (12) | 5 (29) | 2 (13) | 2 (11) | 2 (12) | 0.48 |
| **Kyphoscoliosis (%)** | 0 | 0 | 0 | 0 | 0 | 2 (12) | 0.06 |
| **NAFLD (%)** | 0 | 0 | 1 (6) | 0 | 2 (11) | 1 (6) | 0.41 |
| **NMD (%)** | 5 (26) | 1 (6) | 0 | 2 (13) | 0 | 2 (12) | 0.06 |
| **MND (%)** | 2 (11) | 1 (6) | 1 (6) | 0 | 0 | 3 (18) | 0.28 |
| **Pulmonary HTN (%)** | 0 | 2 (12) | 1 (6) | 2 (13) | 1 (6) | 0 | 0.45 |
| **Schizophrenia (%)** | 1 (5) | 0 | 0 | 0 | 1 (6) | 0 | 0.59 |
| **Stroke (%)** | 0 | 2 (12) | 0 | 0 | 1 (6) | 3 (18) | 0.12 |
| **T2DM (%)** | 1 (5) | 4 (24) | 6 (33) | 6 (38) | 11 (61) | 3 (18) | **0.007** |
| **Relevant Medication** | | | | | | | |
| **Beta-blocker (%)** | 0 | 0 | 4 (22) | 2 (13) | 4 (22) | 1 (6) | 0.08 |
| **Levothyroxine (%)** | 0 | 0 | 1 (7) | 1 (7) | 2 (14) | 1 (6) | 0.50 |
| **LAMA (%)** | 1 (5) | 0 | 2 (11) | 2 (13) | 5 (28) | 7 (41) | **0.010** |
| **LABA** | 0 | 1 (6) | 3 (17) | 2 (13) | 5 (28) | 8 (47) | **0.004** |
| **Another rate limiting agent** | 0 | 0 | 0 | 1 (6) | 0 | 1 (6) | 0.49 |
| **Original Sleep study data** | | | | | | | |
| **ESS** | 10.25 ± 5.12 | 8.36 ± 6.13 | 10.55 ± 6.47 | 9.43 ± 6.13 | 9.09 ± 6.52 | 4.89 ± 6.29 | 0.42 |
| **AHI (events/hr)** | 1.42 ± 0.98 | 23.01 ± 16.29 | 39.34 ± 32.96 | 43.96 ± 24.14 | 5.59 ± 4.42 | 12.63 ± 24.33 | **<0.001** |
| **ODI (events/hr)** | 1.42 ± 0.98 | 23.78 ± 14.57 | 44.56 ± 33.63 | 55.86 ± 22.92 | 10.13 ± 7.05 | 19.34 ± 31.18 | **<0.001** |
| **Mean saturations (%)** | 94.15 ± 1.47 | 91.82 ± 1.14 | 86.86 ± 4.30 | 82.46 ± 5.43 | 83.63 ± 4.24 | 84.78 ± 5.94 | **<0.001** |
| **Time <90%** | 1.26 ± 2.97 | 12.64 ± 8.47 | 62.80 ± 25.31 | 88.44 ± 15.05 | 89.13 ± 18.34 | 72.09 ± 28.41 | **0.012** |
| **Capillary blood gas (if done)** | | | | | | | |
| **pH** | 7.42 ± 0.02 | 7.42 ± 0.02 | 7.41 ± 0.039 | 7.40 ± 0.03 | 7.39 ± 0.03 | 7.40 ± 0.03 | 0.122 |
| **CO2** | 5.03 ± 0.32 | 4.89 ± 0.40 | 5.32 ± 0.31 | 6.65 ± 0.80 | 6.52 ± 0.85 | 6.56 ± 0.61 | **<0.001** |
| **O2** | 10.57 ± 1.44 | 10.91 ± 1.10 | 9.00 ± 1.34 | 8.18 ± 1.13 | 8.06 ± 1.03 | 7.92 ± 1.23 | **<0.001** |
| **HCO3** | 24.73 ± 1.43 | 24.13 ± 1.88 | 24.71 ± 2.60 | 28.35 ± 3.89 | 27.00 ± 3.12 | 28.11 ± 1.83 | **<0.001** |
| **BE** | 0.80 ± 1.26 | -0.41 ± 2.19 | 0.30 ± 2.99 | 4.02 ± 3.13 | 2.77 ± 3.60 | 4.21 ± 1.99 | **<0.001** |
| **Type of PAP therapy** | | | | | | | |
| **None (%)** | 19 | 3 (18) | 5 (28) | 1 (6) | 5 (28) | 8 (47) | **<0.001** |
| **CPAP (%)** | 0 | 12 (71) | 11 (61) | 0 | 1 (6) | 0 | **<0.001** |
| **BIPAP Auto (%)** | 0 | 1 (6) | 1 (6) | 2 (13) | 0 | 0 | **<0.001** |
| **NIV (%)** | 0 | 1 (6) | 1 (6) | 13 (81) | 12 (67) | 9 (53) | **<0.001** |
| **Mortality data** | | | | | | | |
| **Alive** | 16 (89) | 15 (94) | 14 (82) | 12 (86) | 14 (82) | 12 (71) | 0.58 |
| **Died** | 2 (11) | 1 (6) | 3 (18) | 2 (14) | 3 (18) | 5 (29) | 0.58 |

Abbreviations: AHI = apnoea-hypopnoea index; BE = base excess; BIPAP = bilevel positive airway pressure; CPAP = continuous positive airway pressure; CO2 = carbon dioxide; COPD = chronic obstructive pulmonary disease; ESS = Epworth Sleepiness Score; HCO3 – bicarbonate; HTN = hypertension; IHD = ischaemic heart disease; LABA = long acting beta agonist; LAMA = long acting muscarinic antagonist; MND = motor neuron disease; NAFLD = non-alcoholic fatty liver disease; NIV = non-invasive ventilation; NMD = neuromuscular disease; O2 = oxygen; ODI = oxygen desaturation index; T2DM = type 2 diabetes mellitus;

*Most of the data was normally distributed. Continuous data presented as mean ± SD and a one-way ANOVA performed. Categorical data was analysed using the Chi squared test.

Initial analysis of this data and mapping the index AHI, percentage of time spent with saturations below 90% and the sample entropy of oxygen saturation we found that patients with nocturnal hypoxia, a high index AHI and eucapnia, had a similarly elevated sample entropy of oxygen saturation and distribution compared to patients with OSA/OHS. This was suggestive that patients with nocturnal hypoxia but eucapnia, were likely in the main to represent early hypoventilation and behave in a similar fashion to those with hypercapnia. Similarly, patients with nocturnal hypoxia, eucapnia and a low index AHI had a reduced sample entropy of oxygen saturation and similar distribution to patients with OHS only. This data suggested that the severity of OSA drives the change seen in sample entropy of oxygen saturation, but also suggested that a reduced AHI with nocturnal hypoxia or lone OHS represents a sicker patient population whose system is disengaged. This is illustrated in Figure S1.

Figure S1: 3-D map of AHI, time spent with saturations below 90% and Sample Entropy of oxygen saturation


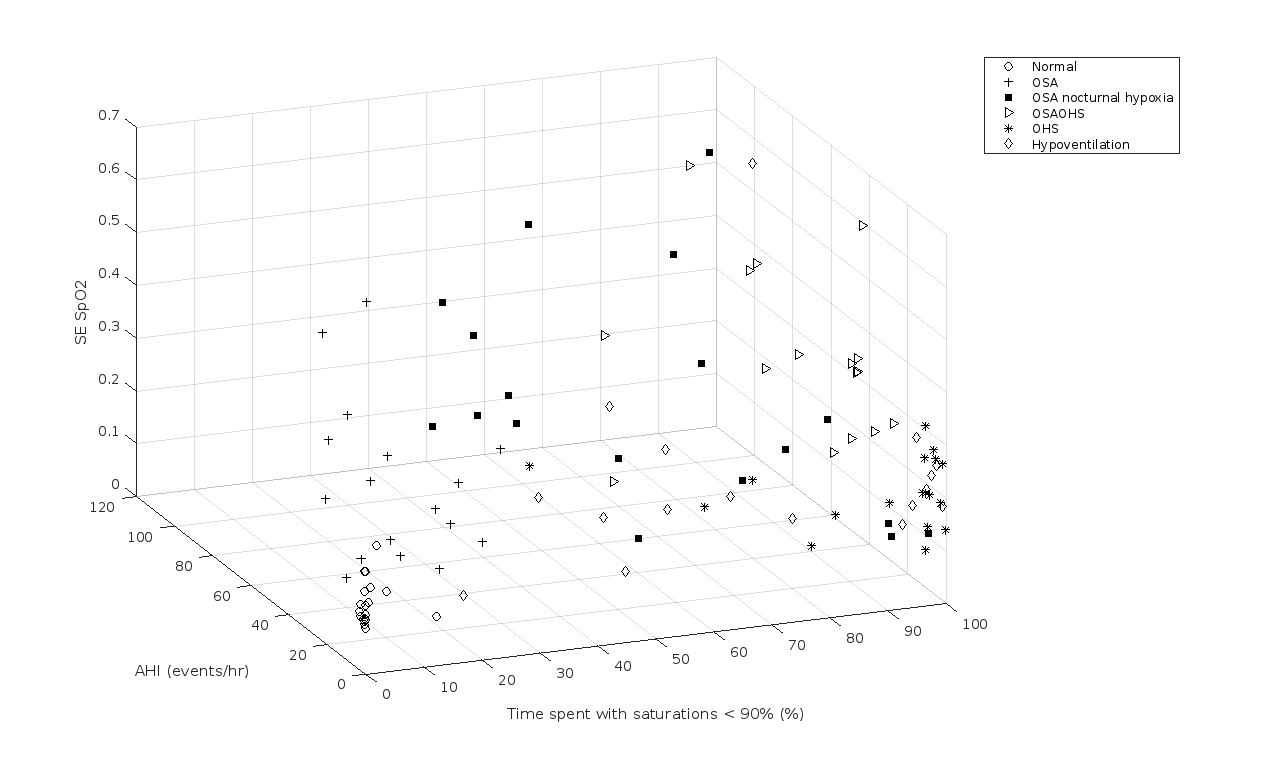


Based on this information, we chose to re-group the 6 groups into 4 clinically relevant groups as outlined in our main paper.

**Training data set**

The training data set had four groups.

Four groups:

1. **Normal –** This was the reference standard. These were multi-channel sleep studies conducted between January 2017 and December 2021 in patients referred to the sleep clinic due to symptoms or suspicion of sleep disordered breathing. The subsequent sleep study was normal and ruled out sleep disordered breathing.
2. **OSA alone (AHI ≥ 5events/hr) –** This is the current, internationally recognised definition of obstructive sleep apnoea.
3. **Sustained nocturnal hypoxia with a high AHI (AHI ≥ 30events/hr and nocturnal hypoxia (time spent with saturation below 90% ≥ 30% (2,3) with or without daytime hypercapnia) –** This group represented patients with severe OSA with sustained nocturnal hypoxia.
4. **Sustained nocturnal hypoxia with a low AHI (AHI < 30events/hr and nocturnal hypoxia (time spent with saturation below 90% ≥ 30% with or without daytime hypercapnia) –** This group represented patients who were potentially a sicker cohort who may be akin to patients with hypoventilation

**Test data set**

The test data set compromised of another group of patients who underwent domiciliary sleep studies between January 2017 and December 2021 who were categorized in the same four groups as the test data set.

The graphical flowchart for patient recruitment can be seen in Figure S2.

Figure S2: Graphical flowchart for patient recruitment

**
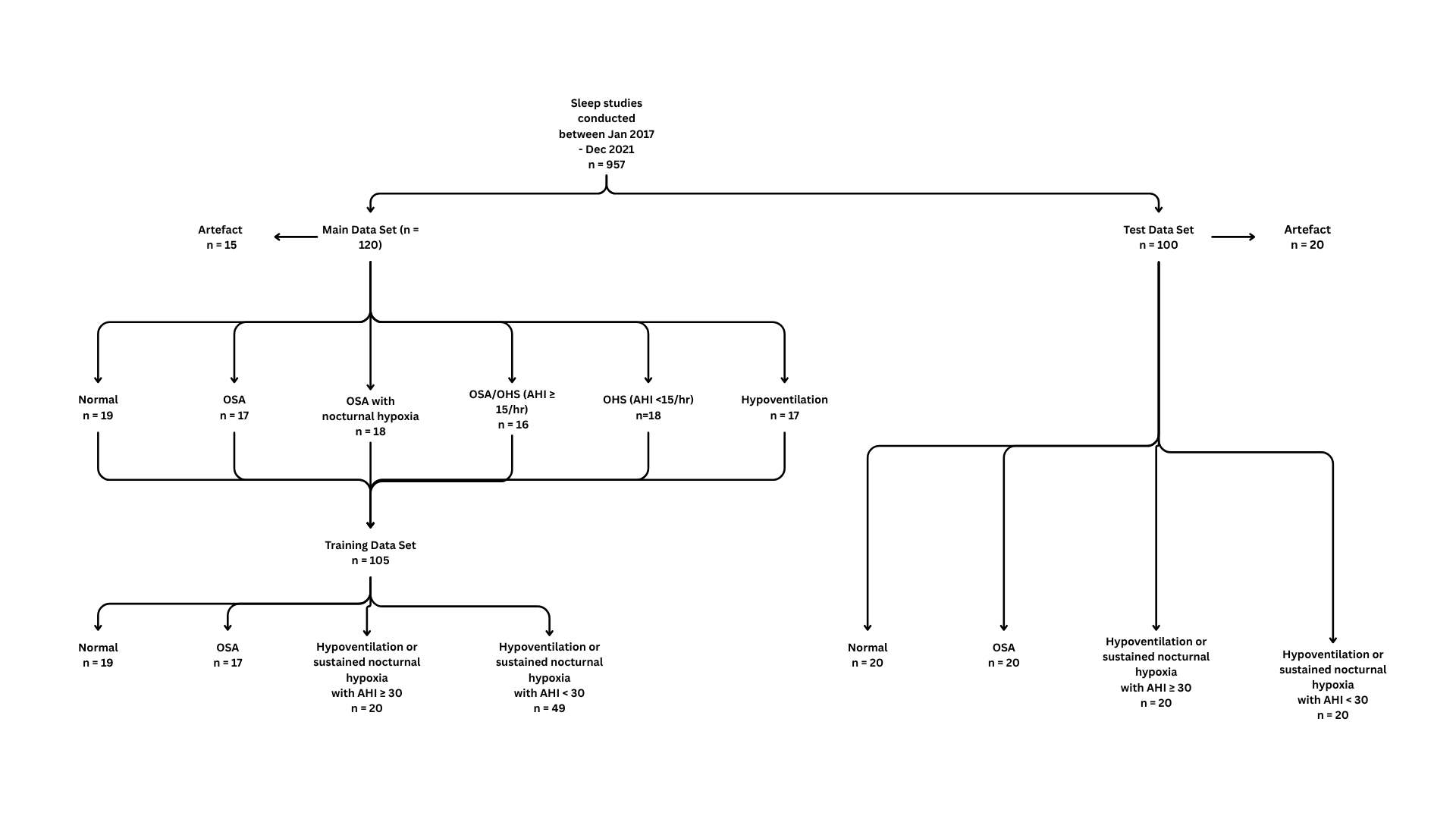
**

**Data Extraction and Analysis**

Cardiorespiratory polygraphies are multi-channel level 3 sleep studies which are routinely used for the diagnosis of sleep disordered breathing and recognised by the American Association of Sleep Medicine as an acceptable reference standard. (4) They are usually manually analysed using Natus® Embla® RemLogic™ diagnostic software. The index sleep studies were exported from this software and uploaded to MATLAB (MathWorks). The original sampling rates for the four main channels of interest are shown below:

- Nasal Flow (NF) – 250 per second
- Respiratory Rate (RR) – derived from nasal flow and not directly measured
- Heart Rate (HR) – 3 per second
- Oxygen Saturation (SpO2) – 3 per second

To perform standardised calculations, these four channels were resampled to 1Hz (i.e., one recording per second). Internationally four hours constitutes enough data for a valid sleep study. To ensure uniformity, the first hour of every sleep study was not analysed, and the subsequent four hours of data (14400 data points) were extracted and analysed. To analyse the data, the data was first cleaned to remove potential artefact in the following traces:

- RR – any measurement above 30 breaths per minute was assumed to be inaccurate data capture and this measurement was changed to the median RR.
- HR – any measurement below 40 beats per minute, was assumed to be inaccurate data capture, and this measurement was changed to median HR.
- SpO2 – any measurement below 50% was assumed to be inaccurate data capture, and this measurement was changed to the median SpO2.

The percentage of artefact for each trace was noted and if a recording had ≥ 10% of aberrant data, that study was not included in the final analysis.

Following this we calculated the mean, standard deviation, and sample entropy for all four channels using well described algorithms in MATLAB. Sample Entropy was calculated using MATLAB codes freely available on PhysioNet (5, 6) with *m* set at 2 and *r* at 0.2 as previously described. (7) Transfer Entropy was calculated using well established MATLAB algorithms to determine the transfer of information between all four signals, thus giving a network map with 16 interactions (4x4). The time lag used for the calculation was 5 seconds (e.g., how does the previous 5 seconds of SpO2 affect the next 5 seconds of HR). This choice is based on previous studies that indicated the Transfer Entropy between cardiorespiratory signals increases with the time lag and reaches a plateau at a time lag of 5-10s. (8)

**Cut-off values from ROC curves**

The main algorithm that was created by analysing the training data set was validated using specific cut-off values of Sample Entropy of oxygen saturations. This was to diagnose OSA compared to normal sleep studies and to diagnose severe OSA/hypoventilation compared to non-severe OSA / hypoventilation.

The cut-off values chosen were designed to be clinically meaningful. For a metric to be clinically useful in differentiating OSA from normal studies, a high sensitivity is required, so that the false negative rates are low and people with OSA are not missed. In view of this, the trial group chose a cut-off value with a high sensitivity (100%) that was still 74% specific.

To differentiate severe OSA/hypoventilation from non-severe OSA/hypoventilation a high specificity is required such that the false positive rates are low and people who have a positive test (i.e., severe OSA/hypoventilation) are appropriately treated with CPAP, but given the low false positive rates with a specific test, patients with non-severe OSA/hypoventilation are not inadvertently given CPAP. Therefore, the trial group chose a cut-off value that has a high specificity (92%) that was still 70% sensitive.

**References**

1. Masa JF, Benítez I, Sánchez-Quiroga M, Gomez de Terreros FJ, Corral J, Romero A, Caballero-Eraso C, Alonso-Álvarez ML, Ordax-Carbajo E, Gomez-Garcia T, González M, López-Martín S, Marin JM, Martí S, Díaz-Cambriles T, Chiner E, Egea C, Barca J, Vázquez-Polo FJ, Negrín MA, Martel-Escobar M, Barbé F, Mokhlesi B. Long-term Noninvasive Ventilation in Obesity Hypoventilation Syndrome Without Severe OSA: The Pickwick Randomized Controlled Trial. *Chest* 2020; 158: 1176-1186.
2. Lewis CA, Fergusson W, Eaton T, Zeng I, Kolbe J. Isolated nocturnal desaturation in COPD: prevalence and impact on quality of life and sleep. *Thorax* 2009; 64: 133-138.
3. Mandal S, Suh ES, Boleat E, Asher W, Kamalanathan M, Lee K, Douiri A, Murphy PB, Steier J, Hart N. A cohort study to identify simple clinical tests for chronic respiratory failure in obese patients with sleep-disordered breathing. *BMJ Open Respiratory Research* 2014; 1: e000022.
4. AASM. The AASM Manual for the Scoring of Sleep and Associated Events: American Academy of Sleep Medicine; 2019.
5. Goldberger AL, Amaral LA, Glass L, Hausdorff JM, Ivanov PC, Mark RG, Mietus JE, Moody GB, Peng CK, Stanley HE. PhysioBank, PhysioToolkit, and PhysioNet: components of a new research resource for complex physiologic signals. *Circulation* 2000; 101: E215-220.
6. Lake DE, Richman JS, Griffin MP, Moorman JR. Sample entropy analysis of neonatal heart rate variability. *Am J Physiol Regul Integr Comp Physiol* 2002; 283: R789-797.
7. Richman JS, Moorman JR. Physiological time-series analysis using approximate entropy and sample entropy. *Am J Physiol Heart Circ Physiol* 2000; 278: H2039-2049.
8. Morandotti C, Wikner M, Li Q, Ito E, Oyelade T, Tan C, Chen P-Y, Cawthorn A, Lilaonitkul W, Mani AR. Decreased cardio-respiratory information transfer is associated with deterioration and a poor prognosis in critically ill patients with sepsis. *Journal of Applied Physiology* 2025; 138: 289-300.
